# MAPK-dependent JA and SA signalling in *Nicotiana attenuata* affects plant growth and fitness during competition with conspecifics

**DOI:** 10.1186/1471-2229-12-213

**Published:** 2012-11-13

**Authors:** Stefan Meldau, Lynn Ullman-Zeunert, Geetha Govind, Stefan Bartram, Ian T Baldwin

**Affiliations:** 1 Department of Molecular Ecology, Max Planck Institute for Chemical Ecology, Hans-Knöll-Str.8, Jena, D-07745, Germany; 2 Current address: Department of Molecular Genetics, Leibniz Institute of Plant Genetics and Crop Plant Research, Corrensstr.3, Gatersleben, D-06466, Germany; 3 Department of Bioorganic Chemistry, Max Planck Institute for Chemical Ecology, Hans-Knöll-Str.8, Jena, D-07745, Germany

**Keywords:** Fitness costs, Induced defense, MAPK, Herbivory, *Nicotiana attenuata*, Salicylic acid, Jasmonic acid, Ethylene, Nitrogen, Photosynthesis

## Abstract

**Background:**

Induced defense responses to herbivores are generally believed to have evolved as cost-saving strategies that defer the fitness costs of defense metabolism until these defenses are needed. The fitness costs of jasmonate (JA)-mediated defenses have been well documented. Those of the early signaling units mediating induced resistance to herbivores have yet to be examined. Early signaling components that mediate herbivore-induced defense responses in *Nicotiana attenuata*, have been well characterized and here we examine their growth and fitness costs during competition with conspecifics. Two mitogen-activated protein kinases (MAPKs), salicylic acid (SA)-induced protein kinase (SIPK) and wound-induced protein kinase (WIPK) are rapidly activated after perception of herbivory and both kinases regulate herbivory-induced JA levels and JA-mediated defense metabolite accumulations. Since JA-induced defenses result in resource-based trade-offs that compromise plant productivity, we evaluated if silencing *SIPK* (ir*SIPK*) and *WIPK* (ir*WIPK*) benefits the growth and fitness of plants competiting with wild type (WT) plants, as has been shown for plants silenced in JA-signaling by the reduction of *Lipoxygenase 3* (*LOX3*) levels.

**Results:**

As expected, ir*WIPK* and LOX3-silenced plants out-performed their competing WT plants. Surprisingly, ir*SIPK* plants, which have the largest reductions in JA signaling, did not. Phytohormone profiling of leaves revealed that ir*SIPK* plants accumulated higher levels of SA compared to WT. To test the hypothesis that these high levels of SA, and their presumed associated fitness costs of pathogen associated defenses in ir*SIPK* plants had nullified the JA-deficiency-mediated growth benefits in these plants, we genetically reduced SA levels in ir*SIPK* plants. Reducing SA levels partially recovered the biomass and fitness deficits of ir*SIPK* plants. We also evaluated whether the increased fitness of plants with reduced SA or JA levels resulted from increased nitrogen or CO_2_ assimilation rates, and found no evidence that greater intake of these fitness-limiting resources were responsible.

**Conclusions:**

Signaling mediated by WIPK, but not SIPK, is associated with large fitness costs in competing *N. attenuata* plants, demonstrating the contrasting roles that these two MAPKs play in regulating the plants’ growth-defense balance. We discuss the role of SIPK as an important regulator of plant fitness, possibly by modulating SA-JA crosstalk as mediated through ethylene signaling.

## Background

Plants have evolved effective defense strategies to ward off natural enemies, including pathogens and herbivores. Allocation of fitness-limiting resources to anti-pathogen and anti-herbivore resistance frequently imposes costs on plants, which are readily seen as reductions in plant growth and fitness. These fitness costs of defense production play a fundamental role in most plant defense theories (reviewed in [1]). Instead of producing costly defense metabolites permanently, plants often activate defense pathways only in response to signals that implicate the presence of attackers. Such plastic defense pathways, so called induced defenses, are generally believed to have evolved as a resource-saving strategy (reviewed in [2]).

Fitness costs of induced resistance pathways are frequently evaluated by manipulating defense hormone levels, such as jasmonic acid (JA) and salicylic acid (SA), two hormones which respectively regulate major anti-herbivore and anti-pathogen defense responses (reviewed in [2-4]). SA mediates plant resistance to biotrophic, hemibiotrophic pathogens and some piercing/sucking herbivores [5]. Priming of SA-related defense responses increases disease resistance and plant fitness in the field [6]. However, under pathogen-free conditions, maintaining the SA-pathway imposes a trade-off for plant growth and fitness when compared to plants with genetically reduced SA levels [7].

The jasmonate signaling cascade, including the wound hormone JA-isoleucine (JA-Ile), is widely considered to be a master regulator of plant resistance to arthropod herbivores as well as various pathogens (reviewed in [8]). Fitness costs imposed by the activation of JA-mediated defense pathways have been measured by treating plants with JA or by using plants altered in JA production or perception. Application of JA and SA reduces seed production and mutants with reduced sensitivity to these hormones tend to have higher fitness correlates in *Arabidopsis thaliana* grown under controlled conditions in a glasshouse experiment [9]. When native populations of Coyote tobacco (*Nicotiana attenuata*) plants were treated with JA, the JA-mediated resistance traits proved to be costly for seed production in the absence of herbivore attack, but benefited plant fitness when plants were attacked by herbivores [10].

Upon herbivore or pathogen attack, endogenous SA and JA levels are strongly regulated by upstream signaling units that mediate defense responses to various attackers. To understand if the ability to be inducible *per se* can result in fitness costs, we need to analyze the trade-offs in biomass and fitness correlates associated with the signaling units upstream of these phytohormone pathways. Following this approach, in *A. thaliana*, a single *R* gene (*RPM1*), which is involved in bacterial pathogen recognition, was demonstrated to result in large fitness costs to plants grown in the field [11]. Similarly, it was shown that natural variation at a single genomic locus, involved in regulating SA and JA levels, can explain growth and resistance phenotypes of a large number of *A. thaliana* accessions [12]. Therefore, analyzing costs of such upstream regulators can help explain growth and defense polymorphism in natural populations. While these studies describe costs of *R* genes involved in resistance to pathogens, the costs of perception and signaling units mediating resistance to herbivores upstream of hormonal sectors remain unexplored.

**Figure 1:**
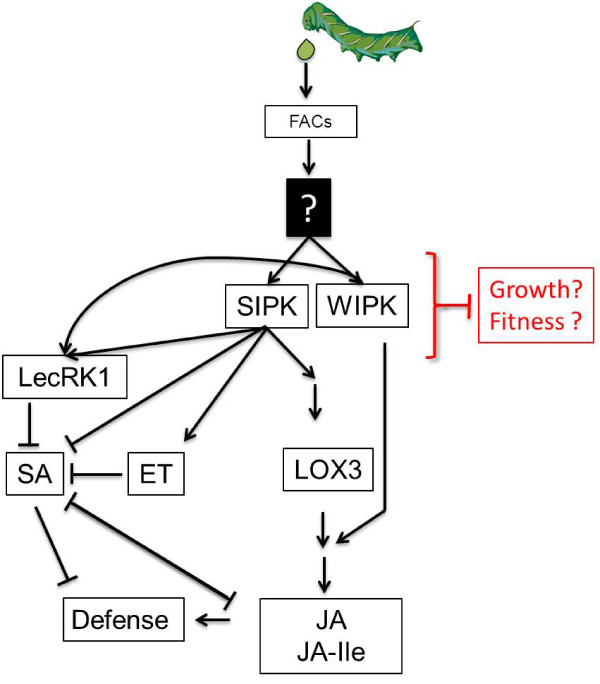
**Herbivory-induced signaling in *N. attenuata.*** Oral secretions of *Manduca sexta* contain fatty acid-amino acid conjugates (FACs), which are perceived by *Nicotiana attenuata* through an unknown perception event (black filled box with question mark), leading to the activation of salicylic acid-induced protein kinase (SIPK) and wound-induced protein kinase (WIPK). SIPK and WIPK regulate transcripts of LecRK1, which is a negative regulator of SA. SIPK and WIPK regulate biosynthesis of jasmonic acid (JA) and its isoleucine conjugate (JA-Ile). SIPK, but not WIPK, also regulates ethylene (ET) emissions, possibly leading to suppressed SA levels, thereby allowing unfettered JA/JA-Ile-mediated defense responses. Growth and fitness consequences of SIPK and WIPK-regulated signaling are unknown (red box).

In *N. attenuata,* one of its main natural defoliators, the lepidopteran larvae *Manduca sexta*, is perceived through fatty acid-amino acid conjugates (FACs) present in the insect’s oral secretions (reviewed in [13]). It was reported recently that FAC perception results in growth reductions in *N. attenuata*[14], but the underlying mechanisms remain elusive. One of the earliest molecular events in FAC perception is the activation of mitogen-activated protein kinases (MAPK, [15]). MAPK activity is important for the induction of plant defense responses upon herbivore attack, including the regulation of various hormonal pathways (Figure 1). In *N. attenuata*, salicylic acid-induced protein kinase (SIPK) and wound-induced protein kinase (WIPK), as well as their homologues in tomato (*Lycopersicum esculentum*), cultivated tobacco (*N. tabacum*) and *A. thaliana* mediate the activation of defense-related hormonal responses in herbivory-induced tissues [15-18]. Both, SIPK and WIPK, regulate wound and herbivory-induced JA and JA-Ile levels, whereas only SIPK regulates herbivory-induced ethylene (ET) levels in *N. attenuata*[15]. LecRK1, which is an important negative regulator of herbivory-induced SA levels is also regulated by SIPK and WIPK ([19]; Figure 1).

*N. attenuata* is an annual plant that grows in the immediate post-fire environment in the Great Basin Desert (Utah, USA) where it occurs in monoculture-like populations, surrounded by conspecific competitors. Because this environment is characterized by highly reduced nitrogen availability, synchronized seed germination and intense intra-specific competition, it represents the primordial agricultural niche. In such transiently resource-rich environments, plants are strongly selected for competitive abilities which depend on maximizing the acquisition and the efficient use of acquired resources. In other words, plants are selected to maximize, and not to optimize, resource acquisition. In this manuscript, we analyzed the costs of maintaining and activating herbivory-induced signaling pathways when plants are grown under the intense resource competition conditions that the plants commonly germinate into in nature, using *SIPK* and *WIPK*-silenced *N. attenuata* plants.

We grew plants transformed with inverted repeat (ir) constructs for *SIPK* (ir*SIPK*) and *WIPK* (ir*WIPK*) in competition with wild type (WT) plants and analyzed plant growth and fitness parameters, with and without simulated herbivory. Quantifying true plant fitness requires the measurements of reproductive success over multiple generations and is therefore difficult to assess. Here we measured flower and seed capsules numbers of plants competing with each other as parameters to assess the fitness consequences of defense signaling pathways. Our data reveal that although both MAPK-silenced lines accumulated less JA after herbivory, only ir*WIPK* plants benefited from the reduced defensive state with higher biomass and fitness. Ir*SIPK* plants accumulated higher levels of SA and when these plants were crossed with oe*NahG* plants that overexpress bacterial salicylate hydroxylase (NahG), to lower free SA levels, we could partially recover growth and fitness parameters caused by SIPK silencing. Although both kinases are frequently reported to regulate common defense pathways, our data demonstrate that SIPK and WIPK regulate different signaling systems that regulate *N. attenuata*’s physiological reconfiguration after herbivore attack and its resulting fitness parameters.

## Results

### Silencing two herbivory-responsive MAPKs differentially affects plant growth under field and glasshouse conditions

**Figure 2:**
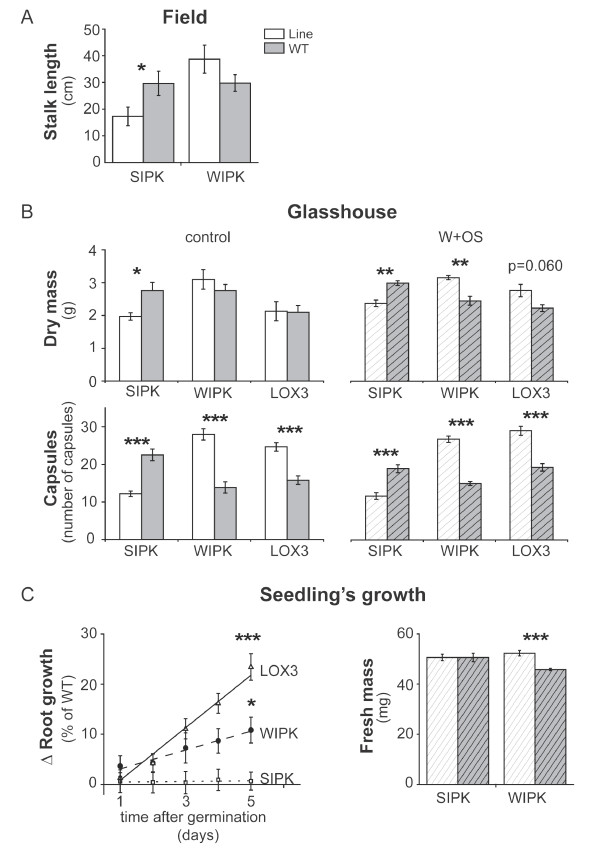
**Growth and fitness of *N. attenuata *plants impaired in herbivory-induced defense signaling.** (**A**) Mean (± SE, n ≥ 9) stalk length of ir*SIPK* and ir*WIPK* plants grown for 44 days in the plant’s natural habitat (Utah, USA) compared to size-matched wild type (WT) plants. (**B**) Mean (± SE) dry mass (n ≥ 3, pooled samples, each containing 5 biological replicates) and total capsule number (n ≥ 19) of transgenic plants (ir*SIPK*, ir*WIPK*) grown in competition with wild type (WT) plants. To simulate herbivory, three rosette leaves of each plant were wounded with a pattern wheel (W) and treated with 10 μL 1:5 diluted *Manduca sexta* oral secretions (OS). Untreated plants served as controls. (**C**) Mean (± SE, n ≥ 16.) relative root growth difference to WT in wounded seedlings grown in competition (asteriks indicate significant differences between ir*LOX3* and ir*WIPK* when compared to ir*SIPK* plants (ANCOVA, F_2,344_ = 13.46, p < 0.001)) and mean (± SE, n = 8) seedling fresh mass grown under low nutrient conditions. Asteriks indicate significant differences between a transgenic line and WT in one pot (Welch two sample *T*-test; ***: p < 0.001; **: p < 0.01; *: p < 0.05).

Two mitogen-activated protein kinases, SIPK and WIPK, in *N. attenuata* have been shown to regulate herbivory-induced defense responses [20]. As defenses are costly and thought to incur trade-offs for plant growth and reproduction [1], we evaluated if silencing *SIPK* and *WIPK* benefited plant growth and fitness. Growth was first analysed in transgenic ir*SIPK* and ir*WIPK* plants in a paired design with WT in their natural habitat in the Great Desert Basin in Utah, USA. Although both transgenic plants have similar reductions in their direct and indirect defenses in comparison to WT plants [20], surprisingly, ir*SIPK* plants were significantly smaller than WT (Figure 2A, Welch two sample *t*-test, p = 0.049), whereas ir*WIPK* plants grew similarly to competing WT plants (Welch two sample *t*-test, p-value = 0.17; Figure 2A). A table with all additional statistical values is provided in the supplemental material (Additional file 1: Table S1).

A similar experiment was carried out in the glasshouse under controlled conditions. Defense-related trade-offs in *N. attenuata* were only found when plants were growing in competition with conspecifics [2]. Thus, we used a paired design of size-matched plants competing for the same resources in individual pots to analyze plant growth and fitness. As MAPK activity and JA-levels are highly induced during herbivore attack [15], we assumed that differences in growth and fitness would be more pronounced when the competing plants were elicited by a simulated herbivory treatment (wouding and application of *M. sexta* oral secretions, W + OS, see Methods). In addition to ir*SIPK* and ir*WIPK*, we used JA deficient plants (as*LOX3*) [20,21] with lower levels of anti-herbivory defense metabolites as “positive controls” as these plants should perform better in comparison to competing WT plants. The results of the glasshouse experiments were comparable with the results from the field. Independent of treatment, ir*SIPK* plants were smaller than WT and produced fewer capsules (Figure 2B), whereas ir*WIPK* plants and as*LOX3* plants produced significantly more dry mass and greater capsule numbers after treatments (Figure 2B). Although MAPK activity and JA levels are highly induced by W + OS treatments, the growth benefits in *WIPK* and *LOX3*-silenced plants were also observed in the absence of W + OS treatments, which suggests that plants are continuously challened by various environmental stresses that activate JA signaling (Figure 2B).

In *N. attenuata*, simulated herbivory can already inhibit growth of seedlings [14]. To assess growth effects of *SIPK* and *WIPK*-silenced plants at the seedling stage, we performed an *in vitro* seedling competition assay (Additional file 2: Figure S1). Under untreated conditions, we did not find growth difference of seedlings (data not shown), whereas wound-induced *WIPK* (ANCOVA, F_5,344_ = 20.79, p = 0.049) and *LOX3* (ANCOVA, F_5,344_ = 20.79, p = 3.72e^-07^) grew faster than WT, while *SIPK*-silenced seedlings grew similarly (ANCOVA, F_5,344_ = 20.79 p = 0.95; Figure 2C). The biomass accumulation of *WIPK*- and *SIPK*-silenced seedlings reflected the trend found in the seedling root growth assay (Welch two sample *t*-test, p = 3.3e-04; Figure 2C). In summary, our data from three different growth assays demonstrated that silencing *WIPK* benefits plant growth and fitness, but that ir*SIPK* plants did not benefit from their JA deficiency.

### Ir*SIPK* plants accumulate more SA in leaf tissues

**Figure 3:**
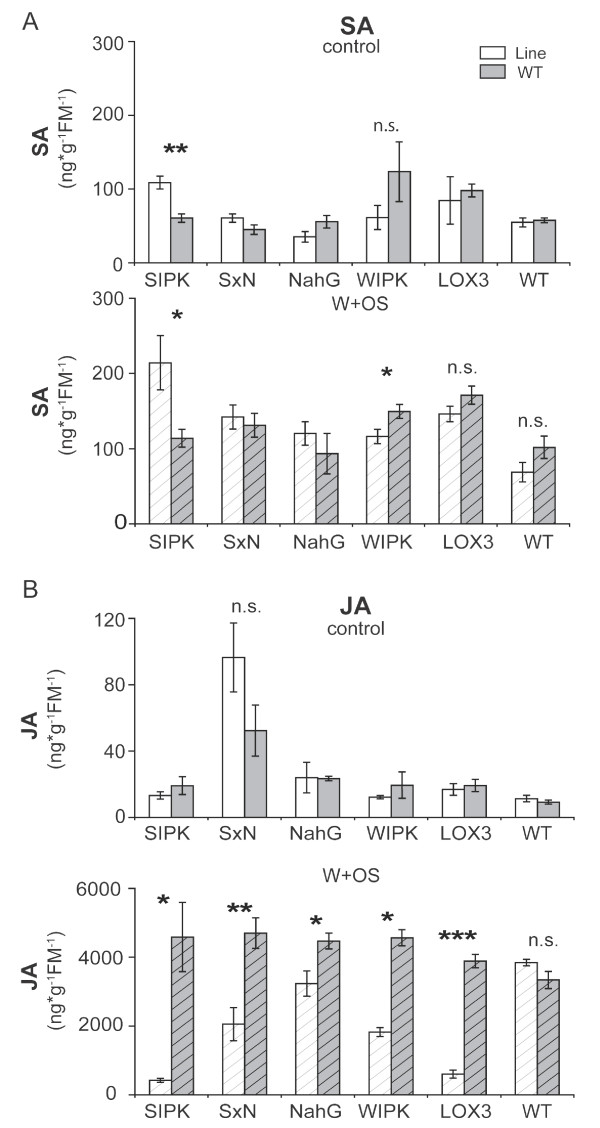
**Phytohormones in leaves of competing *N. attenuata *plants.** Mean (± SE, n ≥ 4) of (**A**) jasmonic acid (JA) and (**B**) salicylic acid (SA) levels of transgenic plants grown in competition with WT plants in 2 L pots. SxN plants are crosses between ir*SIPK* and oeNahG plants. Ten days after transfer to 2 L pots, the youngest source leaf of each plant was harvested as a control and the source-sink transition leaves were wounded with a pattern wheel (W) and treated with 20 μL 1:5 diluted *Manduca sexta* oral secretion (OS) and harvested 1 h after elicitation. Asteriks indicate significant differences between transgenic line and WT in one pot. (Welch two sample *T*-test; ***: p < 0.001; **: p < 0.01; *: p < 0.05).

SA is known to negatively influence plant growth and development [22]. A previous study demonstrated that leaves of ir*SIPK* plants grown in individual pots have higher basal SA-levels than do WT plants (see Supplemental Figure 4 in [23],). To test, if *SIPK*, *WIPK* or *LOX3*-silenced plants also have altered SA-levels when grown in experimental designs that included an intra-specific competitor, SA-levels were measured in untreated leaf tissues and 1 h after W + OS treatments. We found significantly higher SA-levels for ir*SIPK* plants independent of treatment (Welch two sample *t*-test, control: p = 0.002; W + OS: p = 0.048) (Figure 3A). Of the other transgenic lines tested, only ir*WIPK* plants accumulated slightly less SA after W + OS treatments when compared to competing WT plants (Welch two sample *t*-test, p = 0.037). However, JA-levels of ir*SIPK*, ir*WIPK* and ir*LOX3* lines were greatly reduced after W + OS treatments compared to the corresponding WT (Figure 3B). We thus hypothesized that higher SA-levels may influence the growth and fitness phenotype of these JA-deficient plants. To test this hypothesis, ir*SIPK* plants were crossed with an overexpression (oe) salicylic acid hydroxylase (NahG) line (oe*NahG*; [24]). The crossed line, *SxN*, had SA-levels similar to WT (Figure 3A) and lower JA-levels when compared to the corresponding WT plants (Welch two sample *T*-test, p = 0.004; Figure 3B). Notably, JA levels were also reduced in oe*NahG*, when compared to the corresponding WT plants (Figure 3B). A previous report showed that oeNahG plants grown in single pots did not show any difference in JA levels 1 h after W + OS treatments [19] and we hypothesize that different growth conditions in our competition setup might have caused the altered accumulation of JA in the oeNahG line.

### Reducing SA levels in ir*SIPK* plants partially restores the JA deficiency-mediated growth promotion found in JA-deficient plants

**Figure 4:**
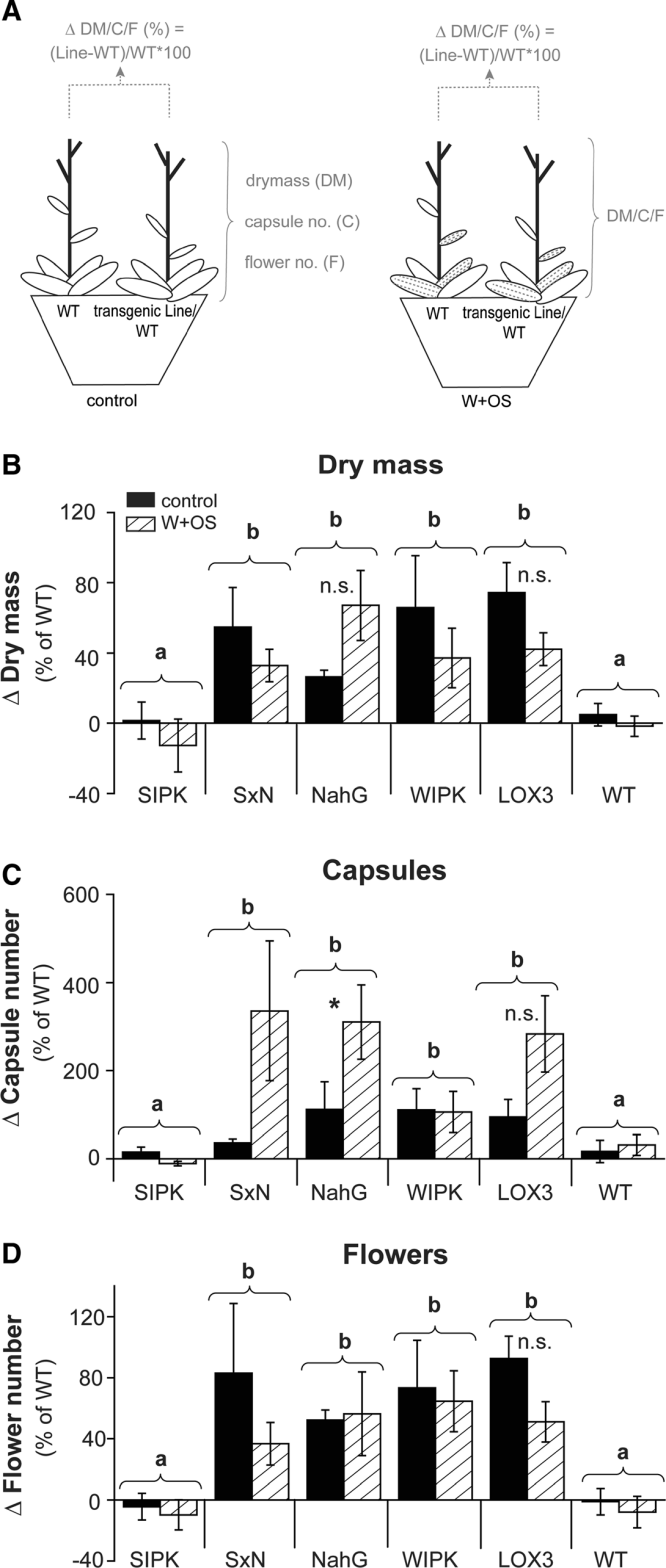
**Reducing SA levels in *SIPK*-silenced plants restores growth.** (**A**) Scheme of the exerimental approach. The transgenic lines (respectively WT plants) were grown with size matched WT plants in competition in one pot. One half of the plants was wounded with a pattern wheel and treated with *Manduca sexta*’s oral secretion (W + OS), the other half was kept as untreated controls. During the experiment, drymass (“DM”), capsule (“C”) and flower (“F”) number were determined. For comparison between treatments, the difference between the two plants (Line-WT) in one pot was calculated for each treatment and expressed in % of the individual WT of that specific pot (see formula in graphic). Differences in (**B**) dry mass, (**C**) capsule number and (**D**) flower number of transgenic lines (ir*SIPK*, ir*WIPK*, ir*LOX3*, *oeNahG*, *SxN*) compared to competing wild type (WT) plants. Ten days after transfer to 2 L pots, rosette leaves of transgenic and WT plants were wounded with a pattern wheel (W) and treated with 10 μL 1:5 diluted *Manduca sexta* oral secretion (OS). Treatment was repeated for two consecutive days. At the stalk elongation stage, W + OS treatment was repeated with expanded S1 leaves. Non-elicited plants were used as controls. The minimum adequate model is represented through small letters (a, b; ANOVA, dry mass: Line: F_2,97_ = 11.12, p < 0.001, capsule number: Line: F_1,58_ = 21.18, p < 0.001, Treatment: F_1,58_ = 8.08, p < 0.01; flower number: Line F_1,58_ = 45.90, p < 0.001). Asterics indicate significant differences between control and W + OS treatment (Welch two sample *t*-test, *: P < 0.05, n.s. = no significant difference).

To investigate the influence of SA on ir*SIPK’s* growth and reproduction, additional competition experiments including *SxN* and oe*NahG* plants were carried out. To combine the results of several experiments in a single graph, we calculated the relative differences between the two competing plants in one pot and expressed them relative to the WT plants used in the individual experiments (Figure 4A). Crossing ir*SIPK* with oe*NahG* resulted in a phenotype similar to plants deficient in JA and JA-mediated defenses (ir*WIPK* and ir*LOX3*). *SxN* plants had greater biomass (ANOVA, F_2,97_ = 11.12, p =4.5e^-05^; Figure 4B), a higher capsule count (ANOVA; Line: F_1,58_ = 21.18, p = 2.32e^-05^; Treatment: F_1,58_ = 8.08, p = 0.006; Figure 4C) and higher number of flowers (ANOVA; Line: F_1,58_ = 45.90, p = 7e^-09^; Figure 4D) than their corresponding WT, whereas ir*SIPK* plants did not, indicating that higher SA levels are fitness-limiting factors in ir*SIPK* plants. Since *SxN* accumulates less JA than oe*NahG* plants, we speculated that the cross would realize greater fitness benefits than the oe*NahG* plants. However, independent of treatment, *SxN* had a similar increase in growth and fitness when compared to their corresponding WT, as did oe*NahG* plants (Figure 4), indicating that silencing *SIPK* impairs growth and fitness in *N. attenuata* also via SA-independent pathways and that *SIPK*-silencing does not effect the *oeNahG*-mediated growth and fitness promotion.

In comparison to the data presented in Figure 2B, where ir*WIPK* plants accumulated significantly more biomass when plants were treated with W + OS, the experiments presented in Figure 4 revealed a constitutively higher biomass in ir*WIPK* plants, when compared to WT plants. In addition, the reduced biomass and seed capsule number is also less pronounced for ir*SIPK* plants, when data from both experiments are compared. These effects could be due to slightly different soil conditions between the two experimental set-ups (see Methods).

### Differences in photosynthetic rates do not explain growth and fitness differences of ir*SIPK*, ir*WIPK* and ir*LOX3* plants

**Figure 5:**
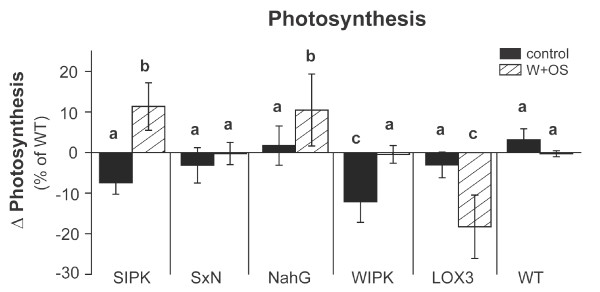
**Growth differences of transgenic plants are not correlated with CO**_**2**_**assimilation rates.** Differences (mean ± SE, n ≥ 4) in photosynthesis rates between transgenic lines (ir*SIPK,*ir*WIPK*, ir*LOX3*, oe*NahG*, *SxN*) compared to competing wild type (WT) plants (calculated as described in Figure 4A). Rosette leaves were OS-elicited as described in Figure 4. Photosynthesis rate was measured at the youngest treated rosette leaf 1 day after the last treatment. The minimum adequate model is represented through small letters (a, b, c; ANOVA, F_2,56_ = 15.70, p < 0.001).

Silencing of ribulose-1,5-bisphosphate carboxylase/oxygenase (RuBisCO) in *N. attenuata* leads to a decrease in photosynthetic rate and reduced plant growth and lower amounts of defense metabolites after treatment with simulated herbivory [25]. Photosynthetic rates are also influenced by herbivory [26], biotic stress [27] and plant hormone levels. In particular exogenous SA application was shown to reduce photosynthetic activity by altering chloroplast structure [28], RuBisCO activity [29] and transcript levels of photosynthetic genes [30]. Thus, we hypothesized, that ir*SIPK* plants would show reduced growth and fitness compared to ir*WIPK,* ir*LOX3* and WT plants as a result of lower photosynthetic rates mediated presumably by its higher SA levels. However, ir*SIPK* plants had a similar or even higher photosynthetic rates than WT and the other transgenic plants (ANOVA, F_2,56_ = 15.7, p = 3.9e^-06^) (Figure 5). Consistent with these results, reduced SA levels in *SxN* plants also did not result in increased photosynthetic rates compared to ir*SIPK* plants. These data indicate that 1) photosynthetic rates under these growth conditions are independent of SA levels and 2) lower amounts of photosynthetic products cannot explain why ir*SIPK* plants did not benefit from reduced JA-mediated defenses.

JA has been shown to down-regulate photosynthesis-related gene expression [21]. Therefore, higher photosynthetic activity might support the growth and fitness of *WIPK* and *LOX3* silenced plants. But ir*WIPK* and ir*LOX3* plants – though they had similar reductions in JA levels compared to WT (Figure 3B) - showed the opposite patterns of photosynthetic rates (Figure 5), and even had a lower photosynthetic activity compared to WT. Therefore, we conclude that the growth promotion of ir*WIPK* and ir*LOX3* plants is not mediated by improved CO_2_ assimilation.

### Leaf JA and SA-levels do not influence competitive ability for nitrogen acquisition

**Figure 6:**
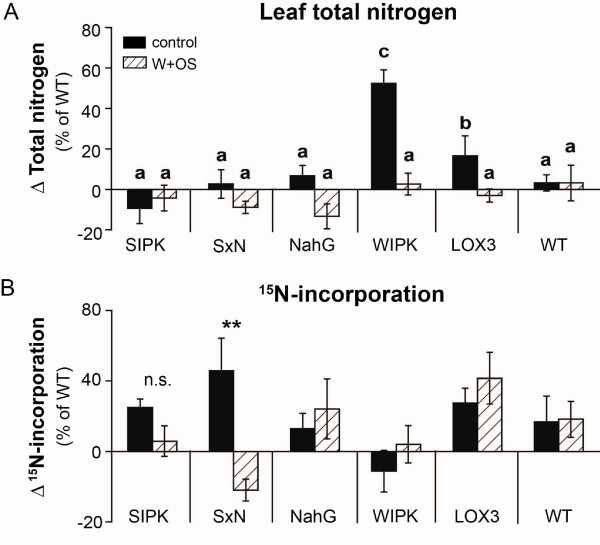
**Growth differences in transgenic plants are not correlated with nitrogen uptake.** Differences (mean ± SE, n ≥ 5) in (**A**) total nitrogen and (**B**) ^15^ N-incorporation between transgenic lines (ir*SIPK*, ir*WIPK*, ir*LOX3*, oe*NahG*, *SxN*) compared to competing wild type (WT) plants (calculated as described in Figure 4A). 7 days after transfer to 2 L pots, the oldest sink leaf was marked and each plant pair was pulse-labeled with 5.1 mg nitrogen delivered as K^15^NO_3 ._ Three days later, rosette leaves of transgenic plants and WT were OS-elicited as described for Figure 4. Leaves were harvested five days after last treatment. Total nitrogen and ^15^ N-incorpration were determined by IRMS (see Methods). The minimum adequate model is represented through small letters (a, b, c; total nitrogen: ANOVA, F_2,102_ = 29.251, p < 0.001;^15^ N-incorporation). Asterics indicate significant differences between control and W + OS treatment (Welch two sample *t*-test, p < 0.01, n.s. = no significant difference).

In addition to photosynthetic rates, the availability of nitrogen influences growth and defense of plants. Under low nitrogen regimes, *N. attenuata* plants grew slower and had lower levels of nitrogen-intensive defense compounds than plants grown under high nitrogen levels [31]. Furthermore, when grown in competition, plants impaired in the production of trypsin proteinase inhibitors (TPIs), a JA-induced nitrogen-intensive defense, produced more seed capsules and were taller than their neighbouring WT plants [32]. Based on these results, ir*SIPK*, ir*WIPK* and ir*LOX3* plants were expected to forgo the costs nitrogen investments in nitrogen-intensive defense metabolites [20,33]. Several lines of evidence suggest that JA [34] and SA [35,36] can influence the plant’s nitrogen assimilation and metabolism. Based on these findings, we evaluated if the differences in growth of the three transgenic lines compared to WT were due to altered competitive availabilities for nitrogen acquisition. We grew the three transgenic lines and WT in competition pairs and pulse-labeled the pots with nitrogen in form of K^15^NO_3_. Although ir*WIPK* and ir*LOX3* plants showed higher total nitrogen content than WT plants before wounding (ANOVA, F_2,102_ = 29.25, p = 9.1e^-11^; Figure 6A), after this treatment they showed a similar nitrogen content as WT. In addition, all transgenic lines incorporated similar amounts of ^15^ N compared to their corresponding WT plants (ANOVA, F_5,94_ = 0.98, p = 0.44; Figure 6B) and only *SxN* showed a treatment effect (Welch two sample *t*-test, p = 0.009). Moreover, seeds of all transgenic lines had similar total N and ^15^ N contents (Additional file 3: Figure S2). Therefore, we conclude that JA and SA levels in leaves do not correlate with nitrogen uptake and content under these growth conditions. However, we cannot exclude that the growth and fitness phenotype of ir*WIPK*, ir*LOX3* and ir*SIPK* was influenced by an altered nitrogen allocation towards growth and reproduction, once the nitrogen was incorporated by the plant.

## Discussion

Activation of MAPKs is one of the earliest molecular events in response to herbivore perception [15]. In this study, a reversed genetics approach was used to analyse if maintaining two herbivory-induced MAPKs, namely NaSIPK and NaWIPK, confer fitness costs to a native tobacco species. Our data show that, although silencing these two MAPKs abolished herbivory-induced JA production, which is known to impose fitness costs on plants, only *WIPK*-silenced plants benefited from these reductions in terms of increased growth and fitness. These results suggest that in addition to JA signaling and JA-associated defenses, SIPK and WIPK regulate different suits of physiological responses after the perception of herbivory, responses that have profound effects on a plant’s ability to maximize their fitness. One of these responses is SA signaling.

### SIPK and WIPK silencing differentially effects SA levels

SIPK and WIPK have frequently been shown to regulate similar responses to biotic and abiotic stresses [37]. Both MAPKs redundantly regulate defense responses and wound and herbivory-induced JA/JA-Ile levels in tomato and *N. attenuata* ([15,16]; [20]). In *N. attenuata*, both kinases regulate transcript levels of genes important for defense against herbivores [15] including LecRK1, which is crucial for herbivory-induced downregulation of SA [19]. Interestingly, only SIPK-, but not WIPK-, silencing led to elevated SA levels; WIPK even accumulated slightly less SA in leaves after simulated herbivory, suggesting that regulation of LecRK1 transcripts is not responsible for the differential accumulations of SA levels (Figure 3B). Silencing *SIPK*, but not *WIPK*, impaires herbivory-induced ET levels in *N. attenuata*. Similarily, only plants deficient in MPK6, the homologue of SIPK in *A. thaliana*, but not MPK3 (WIPK homologue)-deficient plants show reduced herbivory-induced ET levels [15,17]. Diezel and colleagues demonstrated that *N. attenuata* plants impaired in ET biosynthesis or perception accumulated higher levels of herbivory-induced SA; similarily, SA-mediated signaling is suppressed by ET in *A. thaliana *[38,39]. Collectively, these results suggest that the increased SA levels in SIPK silenced plants are a result of impaired ET signaling. Future experiments designed to recover ET emissions in SIPK silenced plants will help to understand the role of ET in mediating the SA phenotype in ir*SIPK* plants.

### Can increased SA mask JA-mediated trade-offs in ir*SIPK* plants?

JA- and methyl-JA-induced responses were reported to negatively affect growth and fitness in several plant species [10,40,41] and plant productivity was enhanced when JA levels or JA/JA-Ile sensitivity were genetically reduced ([9], [42]). Reducing JA levels also increased plant growth and fitness in *WIPK*- and *LOX3*-silenced *N. attenuata* plants (Figures 2 and 4). In contrast, ir*SIPK* plants which showed the highest reductions in herbivory-induced JA levels (Figure 3), did not benefit in terms of growth and fitness. Inhibition of JA-induced defense responses by negative crosstalk through higher SA levels has been intensively studied (reviewed in [43]). In *N. attenuata*, elevated SA levels were found to strongly suppress defense responses to herbivores [19,38]. Although oe*NahG* plants did not show differences in basal SA levels, which is consistant with data presented in [19], the oe*NahG* plants still produced more biomass and fitness when compared to competing WT plants. It is possible that SA levels in other tissues than leaves might be reduced in oe*NahG* plants. Future experiments designed to analyze the SA levels in other tissues, such as roots, might shed light on this phenomenon. By crossing ir*SIPK* with oe*NahG* plants, we tested if higher SA levels could mask the JA deficiency-mediated growth benefits in SIPK silenced plants. Although *SxN* plants showed similar SA levels when compared to oe*NahG*, the cross accumulated significantly less JA (Figure 3). However, the elevated biomass and fitness of oe*NahG* plants was not further increased by JA-deficiency in *SxN*, demonstrating that SA-independent pathways might also be involved in suppressing growth benefits in JA-deficient ir*SIPK* plants. Although we did not observe developmental abnormalities in response to *SIPK*-silencing, these plants might also have other pleiotropic effects, which may influence plant growth and fitness. For example, it was shown that silencing the NaSIPK-homolog MPK6 in Arabidopsis effects stomata patterning [44-46]. In *N. attenuata*, stomata size and density of ir*SIPK* plants are similar to that of WT plants (data not shown). Future studies designed to identify specific phosphorylation targets regulated by SIPK will help to elucidate its important role in plant growth and fitness regulation.

### Mechanisms of JA- and SA-mediated plant growth suppression

Contrasting effects of SA on photosynthesis have been described [7,29,47-49], whereas JA is thought to affect photosynthesis-related gene expression negatively [21]. We did not find a clear correlation between SA or JA levels and photosynthetic rates using our set-up (Figure 5). Since our measurements are just spatiotemporal snapshots, we cannot rule out that photosynthesis and SA or JA levels are correlated at other growth stages or in different tissues.

Another important trait for plant growth under resource-limited conditions is the ability to assimilate nitrogen, a trait that was shown to be altered by JA-treatments in competing *N. sylvestris* plants [50]. However, similar to our photosynthesis measurements, we did not find clear patterns of nitrogen uptake that would explain the growth phenotypes of all JA and SA deficient lines (Figure 6, Additional file 3: Figure S2). Our data do not exclude changes in nitrogen metabolism as a growth promoting factor. All transgenic lines with reduced JA-signaling showed lower levels of nitrogen-intensive defense metabolites than did WT [20,21] which may allocate nitrogen resources towards growth and reproduction. Baldwin [51] discussed fitness optimization as a process of resource allocation and demonstrated that the biosynthesis of nicotine, a JA-induced nitrogen-intensive defense metabolite, can slow growth [52]. JA-induced partitioning of newly fixed carbon and nitrogen into additional secondary metabolite pathways was recently described in *N. tabacum *[53,54] which may lead to an additional reallocation of resources. Further experiments with detailed analysis of different nitrogen pools are required to fully understand the role of nitrogen partitioning in mediating growth and fitness of plants with and without JA and SA perturbations.

In addition to the regulation of metabolite fluxes, SA and JA can also affect developmental processes through the regulation of hormonal pathways. SA can regulate growth through modulation of cell expansion, probably via auxin, [55,56] and might regulate the cell cycle through its crosstalk with cytokinin and brassinosteroid pathways [57,58]. JA was also shown to effect plant growth through the regulation of the cell cycle and cell number in *Arabidopsis* (Zhang et al., [59]). Thus, the alteration of other hormonal pathways might also influence the growth patterns reported here for *N. attenuata* plants with altered MAPK, JA and SA levels .

### Costs of inducibility

We hypothesized that growth and fitness trade-offs imposed by MAPK signaling will only occur when plants were elicited by simulated herbivory since this treatment highly activates SIPK and WIPK. With the exception of *SIPK*-silenced plants, all other transgenic lines, including *LOX3*-silenced and oe*NahG* plants, produced more dry mass and capsules even without simulated herbivory (Figures 2 and 3). These data demonstrate that basal levels of WIPK activity, JA or SA impairs growth and fitness of competing *N. attenuata* plants. In their natural environment, the synchronized germination of *N. attenuata* plants in the first growing season following fires, which in turn results from the detection of smoke-derived germination cues, leads to high intraspecific competition (Baldwin et al. [60]) and our growth setup was designed to capture this natural environmental stress. However, competition with conspecifics may have induced WIPK activity, JA or SA levels in other tissues than leaves, such as their root systems. Therefore the reduced levels of defense traits in other tissues might have caused increased growth and fitness in the control, unelicted plants. Comparing defense traits in different tissues, such as roots, of *N. attenuata* plants grown in single pots with plants in competition will help to answer these questions.

Several studies have demonstrated that herbivore attack changes a plant’s photosynthetic capacity [60-64], and that photosynthetic proteins are commonly downregulated [65]. Our data suggest that the JA and SA mediated costs for growth and fitness are independent of photosynthetic regulation, but we cannot exclude that WIPK activity directly influences photosynthetic activity, as our data have shown lower photosynthesis in unelicited ir*WIPK* plants (Figure 6). Furthermore, ir*SIPK*, ir*LOX3* and ir*WIPK* plants showed a treatment effect on their photosynthetic activity (Figure 5). Therefore, LOX3, SIPK and WIPK activities likely play multiple roles in the regulation of herbivory-induced photosynthesis.

In contrast to their photosynthetic rates, control ir*WIPK* plants as well as ir*LOX3* plants had significantly higher total nitrogen contents in their rosette leaves compared to their corresponding WT plants (Figure 6). These findings indicate, that costs of basal levels of WIPK and LOX3 activity may be amortized by increases in nitrogen resources.

The life history of *N. attenuata* plants may necessitate basic levels of SA, JA and WIPK activity, which come with the cost of reduced growth and capsule production. WIPK and JA-mediated defenses are elicited by attack from the multitude of herbivores that feed on this plant in nature and these defenses use fitness-limiting resources for their production. However, the importance of SA-mediated defense responses in *N. attenuata* are only poorly understood. Our study suggests that maintaining the SA sector must play an important role for fitness of *N. attenuata* not only by moderating JA induced responses, and that SIPK joins two other components shown to suppress SA responses during OS elicitation, response that allow for unfettered JA-mediated defense production: the ethylene burst [38] and LecRK1 [19]. Analyzing the performance of *N. attenuata* plants with different levels of SA under natural conditions are needed to identify the fitness enhancing factors that require the clearly costly SA pathway.

## Conclusions

In this study, we analyzed the fitness consequences of maintaining signaling elements that mediate early herbivory-induced defense responses in native tobacco, *Nicotiana attenuata*. Our data demonstrate that silencing two herbivory-induced *MAPKs*, *NaSIPK* and *NaWIPK*, strongly diminished JA levels, but only *NaWIPK*-silenced plants benefited from these reduced defense responses with increased growth and fitness levels during our competition experiments. We demonstrate that *irSIPK*-plants do not realize the fitness benefits that are commonly enjoyed by JA-deficient plants, partially because Na*SIPK*-silencing leads to higher levels of SA. Photosynthesis and nitrogen acquisition rates cannot explain the growth differences in our setup, indicating that the observed growth phenotypes are rather mediated by resource allocations or signaling mediated growth reductions. Future experiments are needed to identify the specific metabolic pathways by which SA- and JA-signaling divert resources from growth and reproduction. For this analysis identifying the other regulatory targets of SIPK will be essential. Herbivory-induced MAPK activity and JA signaling was shown to vary in natural accessions of *N. attenuata*[66-68] and the natural variation in the SA pathway is currently being analyzed. Determining the costs of the MAPK, JA and SA-mediated pathways for plant growth and fitness contributes to our understanding of the ecological mechanisms behind the genetic variation in these induced defense signaling systems.

## Methods

### Plant growth conditions

#### Germination

Wild-type *Nicotiana attenuata* Torr. Ex. Watson seeds of the 30^th^ (field and first glasshouse experiment, Figure 1) and 31^th^ (other experiments) inbred generations of an accession which originated from seeds that were collected at the Desert Inn Ranch in Utah 1988 [52] and seeds of different transgenic lines, were sterilized and germinated on Gamborg’s 5 media according to Kruegel et al. [69]. For each of the constructs, several independently transformed, homozygous lines harboring single insertions with similar phenotypes, are available and have been fully characterized. For practical reasons, we only used one of the previously described ransgenic lines. The transformed lines used in this study have been previously characterized in the following publications: ir*SIPK* (A-109) and ir*WIPK* (A-56) were described in [20]; oe*NahG* (A-481) in [24,70] and [19]; as*LOX3 *(A-300) in [21]; ir*LOX3* (A-562), in [71]. After using as*LOX3* plants in the first experiments, we used newly generated ir*LOX3* plants, because of their more pronounced reduction in JA levels (22-50% reduction of the OS-elicited JA burst in as*LOX3 *[21], 81-83% in ir*LOX3* ([71], as compared to WT plants).

#### Glasshouse

For glasshouse experiments the plants were transferred to Teku pots ten days after germination. Ten days later the plants were planted into 2 L competition pots. A transgenic plant was always paired with a WT plant. Pots with two WT plants were used as comparison. Plants were grown at 26–28°C under 16 h of light as described by Kruegel et al. [69]. The glasshouse experiment in 2010 was performed with the following modifications: Frühsdorfer Nullerde was used as substrate with additional 0.5 g/L PG Multimix (14, 16 and18 days after transfer to 2 L pots), 0.85 g/L phosphate, 0.05 g/L Micromax (Scotts), 0.35 g/LMgSO_4_7H_2_O added to the soil. As fertilizer, Peters Allrounder (Scotts) was added (20 g/400 L day7-14, 40 g/400 L day 14–21, 15–30 g/400 L after day 21) with an additional amount of Borax (3 g/400 L day 1–7, 2 g/400 L day 7–14, 1 g/400 L after day 14). To perform the experiments under nitrogen-limiting conditions, external nitrogen supplementations were stopped after plants were transferred to 2 L pots in all experiments.

#### Field

Field experiments were carried out as described by Meldau et al. [20]. In brief: seedlings were transferred into hydrated peat pellets fifteen days after germination. After gradual adaption to the local environmental conditions over 14 days, ir*SIPK* and ir*WIPK*, each paired with one size-matched WT plant, were transplanted into an irrigated field plot at the Lytle Ranch Preserve. The release of transgenic plants was carried out under APHIS notification (06-242-101 n). Growth was measured 30 days after transplantation to the field.

### Plant treatment and performance measurements

Eight days after transfer to 2 L competition pots, each plant pair was pulse-labelled with 10.2 mg (5.1 mg ^15^ N and 5.1 mg ^14^ N) nitrogen as KNO_3_ (Chemotrade, Leipzig; Merck). Three days later- giving the plants time to assimilate the labeled nitrogen- the oldest sink leaf, youngest source leaf and transition leaf, were wounded (W) with a pattern wheel and the puncture wounds immediately treated with 10 μL 1:5 diluted *Manduca sexta* oral secretion (OS) (W + OS) over 3 consecutive days in order to simulate continuous herbivore feeding damage. This treatment effectively mimics herbivore attack and allows for uniform induction kinetics [72].

The oldest sink leaf at the time of labeling was harvested 8 days after the first treatment, while samples of untreated plants were used as controls. When all plants were elongated and prior to bud formation (6 days after the last treatment), S1 leaves were wounded and treated as described for rosette leaves. The flowers, both open and closed, and capsules were then counted between day 63 and 65 after germination. The plants were harvested 12 days later and oven dried for 3 days to determine the dry mass.

### *In vitro* seedling growth assay

WT and transgenic lines (ir*WIPK*, ir*SIPK* and ir*LOX3*) of *N. attenuata* used in the glasshouse experiments were used for seedling growth assays. The seeds were sterilized and germinated (Kruegel et al., [69]) on full strength media consisting of H_3_BO_3_ 10 μM, MnSO_4_ 0.5 μM, ZnSO_4_ 0.5 μM, CuSO_4_ 0.1 μM (NH_4_)_6_Mo_7_O_24_ 0.01 μM, Fe-EDTA 15 μM, KH_2_PO_4_ 0.5 mM, MgSO_4_ 1.2 mM, CaCl_2_ 2.0 mM. Nitrogen was supplied as KNO_3_ at a concentration of 2 mM nitrogen [73]. The seedlings were transferred to 1/4^th^ strength media for competition assay when the root length was approximately 1 cm. Special square petri dishes (120 × 120 × 17 mm) for competition experiment were made by cutting the solidified 1/4^th^ strength media into blocks of 1 cm wide with 0.5 cm space between blocks; and approximately upper 2 cm was cut. On each block, wild type seedling was paired with uniform length seedling of either irWIPK or irSIPK or irLOX3 (see also Additional file 2: Figure S1). The shoots were placed in the upper air filled portion of the block. After transfer to blocks, one of the cotyledonary leaves of each seedling was wounded with the tips of bent forceps (making three pin holes). The petri dishes were wrapped with a layer of fabric tape (Micropore 3 M Health Care, Neuss, Germany) to allow for gas exchange and were placed vertically in a growth chamber (Kruegel et al., [69]) to ensure that roots grew to the bottom. The shoots grew in the air filled volume of the upper portion of the blocks. The seedlings were allowed to adjust to the transfer shock for one day after which the growth of roots was monitored on a daily basis. At the end of the 7 day competition experiment, the fresh mass of the roots and shoots of both members of the competing pair was determined: the wild type and its competing transformed line.

### Phytohormone analysis

For phytohormone analysis, plants were grown as described above. The plants were used only for phytohormone analysis and not included into the other analyses. The youngest source leaves were harvested as a control 10 days after transfer to 2 L competition pots, and then transition leaves were wounded and treated with 20 μL 1:5 diluted *M. sexta* oral secretion. The treated leaves were harvested an hour later after removal of the midrib and immediately frozen in liquid nitrogen. After extraction, phytohormones were analysed on an LC-MS⁄MS system (Varian 1200 Triple-Quadrupole-LC-MS system; Varian, Palo Alto, CA, USA according to [17]).

### Photosynthesis measurement

Photosynthesis was measured indirectly by determining CO_2_ assimilation rates. At least 5 replicates were used to analyze photosynthesis using a LI-COR 6400 portable photosynthesis system (LI-COR Bioscience) with 400 μmol/mol CO_2_ concentration and light intensity of 1200 μmol/m^2^/s for measurements.

### Sample preparation for isotope ratio mass spectrometry

^15^ N-incorporation of seed and leaves was analyzed by an elemental analyzer – continuous flow – isotope ratio mass spectrometry (EA–CF–IRMS). One capsule per plant was harvested 71 days after germination. The capsules were harvested when they showed the first signs of opening. Seeds were dried for 2 weeks at room temperature before measurement. The leaf blades of the oldest sink leaf at time point of labeling were harvested 5 days after the last treatment, dried at 60°C for 48 h and homogenized before analysis.

In order to accommodate the high sensitivity of the IRMS, samples were diluted to a final labeling of about 1 atom% ^15^ N by adding a standard (acetanilide; alice-1) to the sample. Seeds weighing 0.3 mg ± 20% (approximately two seeds), were placed in 40 μL tin capsules together with 0.7819 mg ± 20% of the standard. Roughly 0.1250 mg ± 20% of the homogenized and dried plant material was diluted with 0.8325 ± 20% mg of the standard. The exact sample and dilution masses were determined and used to calculate ^15^ N abundance. Three technical replicates of each sample were analyzed.

### Isotope ratio mass spectrometry analysis

The tin capsules were sealed and combusted (oxidation at 1020°C, reduction at 650°C in a constant helium stream (80 mL min^-1^) quantitatively to CO_2_, N_2_ and H_2_O in an elemental analyzer (EuroEA CN2 dual, Hekatech, Wegberg, Germany). After passing a CO_2_/water trap (NaOH/MgClO_4_) and a chromatographic CN-column at 85°C, the remaining N_2_ was transferred via an open split to a coupled isotope ratio mass spectrometer (IsoPrime, Micromass, Manchester, UK). The laboratory working standard was calibrated using IAEA-N-1 reference material with a δ ^15^ N value of +0.43‰. A caffeine standard (cafice-1) was analyzed together with the samples as quality analysis reference material for long-term performance monitoring of the entire analytical procedure (for details see Werner et al., [74]).

Isotopic ratios of nitrogen

(1.3) $$R_{15_{N}}=\frac{\left[{}^{15}N\right]}{\left[{}^{14}N\right]}$$

are expressed in δ notation versus the international standard N_2_(Air) with ^15^R_std_ = 0.0036765.

(1.4) $${\text{δ}}^{15}N_{\text{sa}}=\left(\frac{{}^{15}R_{\text{sa}}{-}^{15}R_{\text{std}}}{{}^{15}R_{\text{std}}}\right)$$

Usually given in ‰ (per mil)

(1.5) $${\text{δ}}^{15}N_{\text{sa}}(‰)=\left(\frac{{}^{15}R_{\text{sa}}}{{}^{15}R_{\text{std}}}-1\right)\cdot 1000$$

Based on the δ notation, isotope abundance ^15^ N (%) was calculated with 1.6.

(1.6) $${}^{15}N_{\text{sa}}(‰)=\frac{100}{\left(1/\left(\frac{{\text{δN}}_{\text{pt}}(‰)}{1000}+\right)\cdot^{15}R_{\text{std}}\right)+1}$$

based on the following equations

(1.7) $${}^{15}N_{\text{sa}}(‰)=\frac{{}^{15}R_{\text{sa}}}{{1+}^{15}R_{\text{sa}}}\cdot 100$$

(1.8) $${}^{15}R_{\text{sa}}=\left(\frac{{\text{δ}}^{15}N_{\text{sa}}(‰)}{1000}+1\right)\cdot^{15}R_{\text{std}}$$

for labeled plant tissue diluted with acetanilide (alice-1, δ^15^N_alice-1_ = −1.36, 10.36% N) calculations were based on the following relations:

(1.9) $${\text{δ}}^{15}N_{\text{pt}}(‰)=\frac{{\text{δ}}^{15}N_{\text{sa}}(‰){-\text{δ}}^{15}N_{\text{alice}-1}(‰)\cdot x_{\text{alice}-1}}{x_{\text{pt}}}$$

(1.10) $$\begin{array}{l} x_{\text{alice}-1}=\frac{\%N_{\text{alice}-1}\cdot m_{\text{alice}-1}}{{\text{totN}}_{\text{alice}-1}{\text{+totN}}_{\text{pt}}}\;\text{and}\;x_{\text{pt}}=\frac{\%N_{\text{pt}}\cdot m_{\text{pt}}}{{\text{totN}}_{\text{alice}-1}{\text{+totN}}_{\text{pt}}}\\ \text{With}\;\%\;N_{\text{pt}}=\frac{\%N_{\text{sa}}\cdot m_{\text{sa}}-\%N_{\text{alice}-1}\cdot m_{\text{alice}-1}}{m_{\text{pt}}} \end{array}$$

With ^15^ N(%) = atom percent of ^15^ N; R_st_ = isotope ratio of standard; alice-1 = acetanilid used for dilution; sa = measured sample; m = mass; %N = total nitrogen percentage; pt = plant tissue sample; totN = total nitrogen mass

### Statistical analysis

All statistical analyses were performed using the software program R (R Developmental Core [75] and the libraries therein (http://www.r-project.org/). For ANOVA analysis, if the assumption of homoscedasticity of variances was violated or the residuals did not follow a normal distribution, the response variables were transformed prior to the analyses using Box-Cox transformation [76]. The Box-Cox-lambda was estimated using Venables’ and Ripley’s MASS library for R. All models were simplified to the minimum adequate model using Akike’s information criterion [77]. The Welch two sample *T*-test was used, in order to account for heteroscedasticity in some data sets. To facilitate comparisons of all statistical analysis, this test was used in all cases.

## Competing interests

The authors declare that they have no competing interests.

## Authors’ contributions

SM and ITB did field experiments; SM and LUZ performed glasshouse experiments; GG and ITB established and GG performed *in vitro* seedling competition assays; SB, SM and L UZ performed the IRMS measurements; SM and LUZ prepared the manuscript, GG, SB and ITB edited the manuscript; LUZ conducted statistical analyses; SM, LUZ, GG and ITB designed experiments. All authors read and approved the final manuscript.

## Supplementary Material

Additional file 1 **Table S1.** Statistical analyses and their P values. Click here for file

Additional file 2 **Figure S1.** Representative pictures of the competition setups. Click here for file

Additional file 3 **Figure S2.** Nitrogen contents of seeds of competing plants. Click here for file
