# Supplementary material for: MAPK-dependent JA and SA signalling in Nicotiana attenuata affects plant growth and fitness during competition with conspecifics
Source: BMC Plant Biol. 2012 Nov 13;12:213. doi: 10.1186/1471-2229-12-213 (PMC3519580; doi:10.1186/1471-2229-12-213)
Supplement: Additional file 1 — Table S1. Statistical analyses and their P values. [file 1471-2229-12-213-S1.docx]

Supplemental table1. Statistical analyses and their P values

|  | **tested parameter^1^** | **statistical test** | **p-value^2^** |
| --- | --- | --- | --- |
| 2a | stalk length SIPK c/os | Welch two sample t-test | 0.049 |
| 2a | stalk length WIPK c/os | Welch two sample t-test | ---- |
| 2b | dry mass iSIPK line/wt c | Welch two sample t-test | 0.028 |
| 2b | dry mass WIPK line/wt c | Welch two sample t-test | ---- |
| 2b | dry mass asLOX3 line/wt c | Welch two sample t-test | ---- |
| 2b | dry mass SIPK line/wt os | Welch two sample t-test | 0.003 |
| 2b | dry mass WIPK line/wt os | Welch two sample t-test | 0.009 |
| 2b | dry mass asLOX3 line/wt os | Welch two sample t-test | 0.060 |
| 2b | capsule no. SIPK line/wt c | Welch two sample t-test | 3.29*10^-06^ |
| 2b | capsule no. WIPK line/wt c | Welch two sample t-test | 6.92*10^-08^ |
| 2b | capsule no. asLOX3 line/wt c | Welch two sample t-test | 4.77*10^-06^ |
| 2b | capsule no. SIPK line/wt os | Welch two sample t-test | 8.07*10^-06^ |
| 2b | capsule no. WIPK line/wt os | Welch two sample t-test | 7.36*10^-14^ |
| 2b | capsule no. as*LOX3* line/wt os | Welch two sample t-test | 1.88*10^-7^ |
| 2c | seedlings’ root growth irLOX3/SIPK | ANCOVA | 3.72*10^-7^ |
| 2c | seedlings’ root growth WIPK/SIPK | ANCOVA | 0.049 |
| 2c | seedlings’ root growth SIPK/WT | ANCOVA | ---- |
| 2c | seedlings’ biomass WIPK c/os | Welch two sample t-test | 3.32*10^-4^ |
| 2c | seedlings’ biomass SIPK c/os | Welch two sample t-test | ---- |
| 3a | SA SIPK line/wt c | Welch two sample t-test | 0.002 |
| 3a | SA SxN line/wt c | Welch two sample t-test | ---- |
| 3a | SA NahG line/wt c | Welch two sample t-test | ---- |
| 3a | SA WIPK line/wt c | Welch two sample t-test | ---- |
| 3a | SA irLOX3 line/wt c | Welch two sample t-test | ---- |
| 3a | SA WT line/wt c | Welch two sample t-test | ---- |
| 3a | SA SIPK line/wt os | Welch two sample t-test | 0.048 |
| 3a | SA SxN line/wt os | Welch two sample t-test | ---- |
| 3a | SA NahG line/wt os | Welch two sample t-test | ---- |
| 3a | SA WIPK line/wt os | Welch two sample t-test | 0.037 |
| 3a | SA irLOX3 line/wt os | Welch two sample t-test | ---- |
| 3a | SA WT line/wt os | Welch two sample t-test | 0.085 |
| 3b | JA | all tests | ---- |
| 3b | JA SxN line/wt c | Welch two sample t-test | ---- |
| 3b | JA SIPK line/wt os | Welch two sample t-test | 0.015 |
| 3b | JA SxN line/wt os | Welch two sample t-test | 0.004 |
| 3b | JA NahG line/wt os | Welch two sample t-test | 0.017 |
| 3b | JA WIPK line/wt os | Welch two sample t-test | 0.026 |
| 3b | JA irLOX3 line/wt os | Welch two sample t-test | 6.19*10^-5^ |
| 3a | JA WT line/wt os | Welch two sample t-test | ---- |
| 4b | dry mass line c/os | ANOVA | 4.5*10^-5^ (Line) |
| 4b | dry mass SIPK c/os | Welch two sample t-test | ---- |
| 4b | dry mass SxN c/os | Welch two sample t-test | ---- |
| 4b | dry mass NahG c/os | Welch two sample t-test | 0.077 |
| 4b | dry mass WIPK c/os | Welch two sample t-test | ---- |
| 4b | dry mass irLOX3 c/os | Welch two sample t-test | ---- |
| 4b | dry mass WT c/os | Welch two sample t-test | ---- |
| 4c | capsule number line c/os | ANOVA | 2.32*10^-5^ (Line) |
| 4c | capsule number line c/os | ANOVA | 0.006 (Treatment) |
| 4c | capsule number SIPK c/os | Welch two sample t-test | ---- |
| 4c | capsule number SxN c/os | Welch two sample t-test | ---- |
| 4c | capsule number NahG c/os | Welch two sample t-test | ---- |
| 4c | capsule number WIPK c/os | Welch two sample t-test | ---- |
| 4c | capsule number irLOX3 c/os | Welch two sample t-test | 0..99 |
| 4c | capsule number WT c/os | Welch two sample t-test | ---- |
| 4d | flower number line c/os | ANOVA | 7.0*10^-9^ (Line) |
| 4d | flower number SIPK c/os | Welch two sample t-test | ---- |
| 4d | flower number SxN c/os | Welch two sample t-test | ---- |
| 4d | flower number NahG c/os | Welch two sample t-test | ---- |
| 4d | flower number WIPK c/os | Welch two sample t-test | ---- |
| 4d | flower number irLOX3 c/os | Welch two sample t-test | 0.082 |
| 4d | flower number WT c/os | Welch two sample t-test | ---- |
| 5 | photosynthesis | ANOVA | 3.85*10^-6^ (Line) |
| 6a | total nitrogen line c/os | ANOVA | 9.09*10^-11^ |
| 6b | ^15^N-incorporation line c/os | ANOVA | ---- |
| 6b | ^15^N-incorporation SxN c/os | Welch two sample t-test | 0.009 |

^1^ Contains first the parameter analyzed than the line and treatment which were tested; SIPK = salicylic acid protein kinase; WIPK = wound induced protein kinase; LOX3 = lipoxygenase 3;c = control; os = wound and oral secretion treatment; / = indicates which treatments were compared

^2^ Only p- values < 0.1 are shown.
