# Supplementary figures and images for: MAPK-dependent JA and SA signalling in Nicotiana attenuata affects plant growth and fitness during competition with conspecifics
Source: BMC Plant Biol. 2012 Nov 13;12:213. doi: 10.1186/1471-2229-12-213 (PMC3519580; doi:10.1186/1471-2229-12-213)

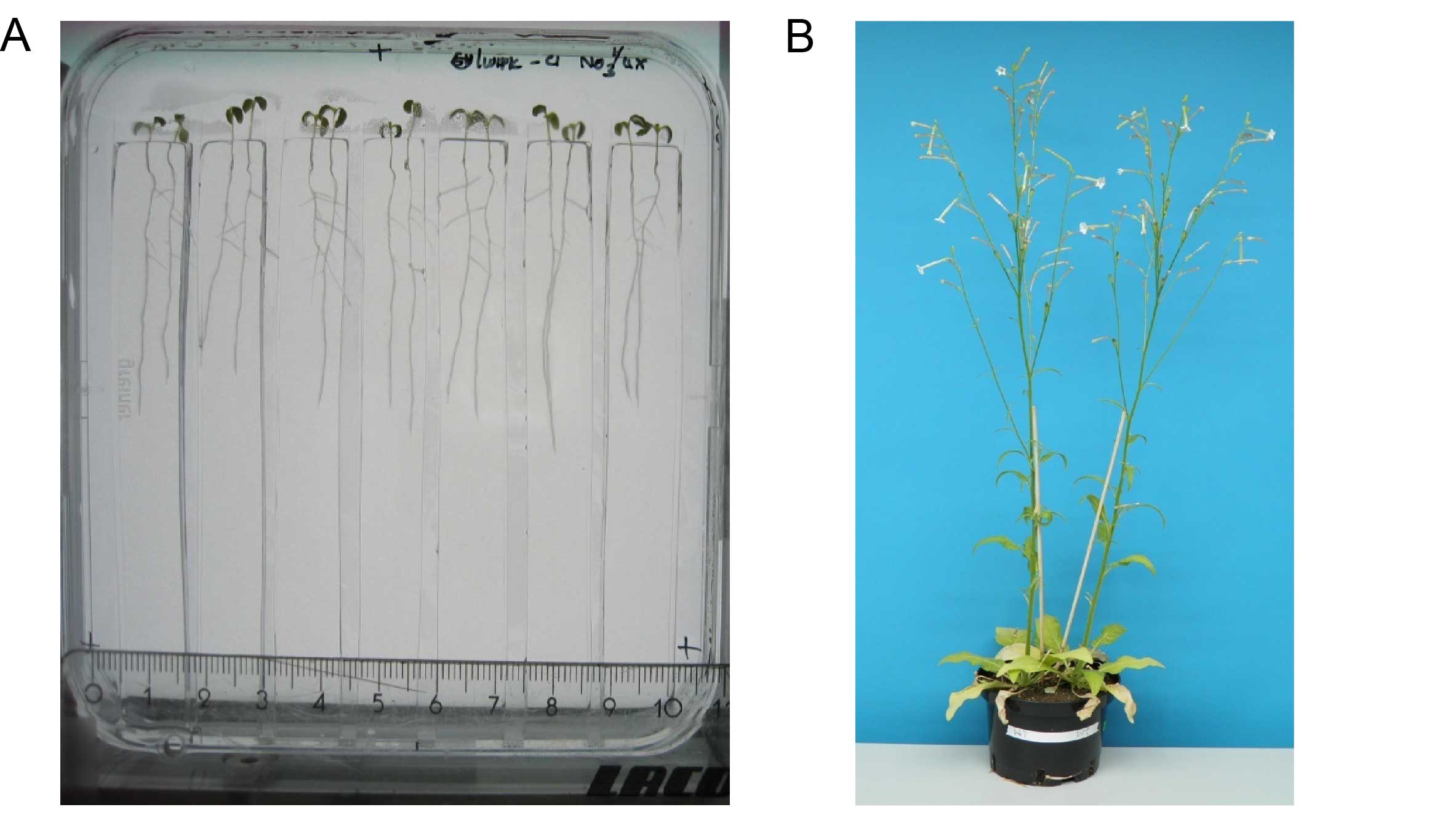

Supplement: Additional file 2 — Figure S1. Representative pictures of the competition setups. [file 1471-2229-12-213-S2.jpeg]

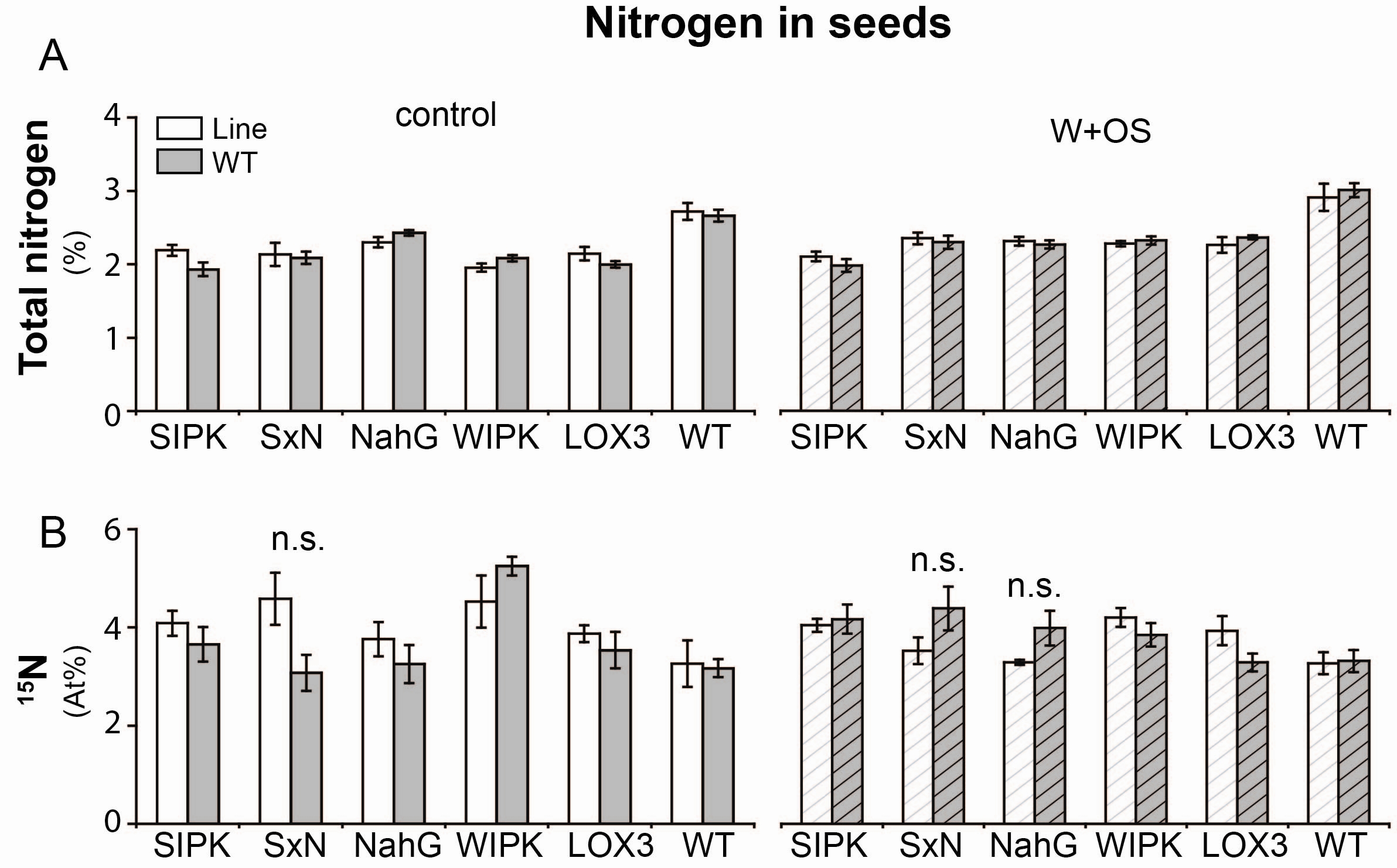

Supplement: Additional file 3 — Figure S2. Nitrogen contents of seeds of competing plants. [file 1471-2229-12-213-S3.jpeg]
